# Supplementary material for: Unified treatment of spin torques using a coupled magnetisation dynamics and three-dimensional spin current solver
Source: Sci Rep. 2017 Oct 11;7:12937. doi: 10.1038/s41598-017-13181-x (PMC5636894; doi:10.1038/s41598-017-13181-x)
Supplement: Supplementary file 1 — Supplementary Information [file 41598_2017_13181_MOESM1_ESM.pdf]

Unified treatment of spin torques using a coupled magnetisation dynamics and three-dimensional spin current solver

Supplementary Information

Serban Lepadatu

Jeremiah Horrocks Institute for Mathematics, Physics and Astronomy, University of Central Lancashire, Preston PR1 2HE, U.K.

Spin Torques in CPP-GMR Stacks

It is well known that both field-like and spin-like torques act on the layers of a current perpendicular to the plane giant magneto-resistance (CPP-GMR) stack [1]. These torques are of the form $a\mathbf{m}\times(\mathbf{m}\times\mathbf{m}_F) + b\mathbf{m}\times\mathbf{m}_F$, where $\mathbf{m}$ is the local magnetisation direction of the free layer, and $\mathbf{m}_F$ is the magnetisation direction of the fixed layer. For a macrospin approximation these torques may be added to the LLG equation with appropriate values for the coupling constants a and b [2]. In general however these coefficients depend on the spin accumulation and have a spatial dependence. Moreover the spin accumulation is important in understanding the magneto-resistance of the CPP-GMR stack [3]. For completeness the model implemented here is tested in a simple spin valve, showing simultaneous reproduction of both the spin torque switching effect, as well as the magneto-resistance effect. The full micromagnetics model is used, including demagnetising and direct exchange contributions, coupled to the three-dimensional spin current solver. The CPP-GMR stack consists of the layering N (59 nm) / F (5 nm) / N (2 nm) / F (3 nm) / N (59 nm). The thicker F layer is the fixed magnetic layer where the magnetisation is kept fixed along the $\boldsymbol{x}$ direction, and the thinner F layer is the free magnetic layer. This is shown in Figure S1, where two extreme configurations are distinguished: the anti-parallel configuration (AP) where the magneto-

resistance is the highest, and the parallel configuration (P) where the magneto-resistance is the lowest. The stack is elliptical in shape with 160 nm × 40 nm dimensions, and the electrodes are placed at the *z*-axis ends of the structure. Stair-step boundary corrections are applied to the elliptical shape to correct for the finite difference artefacts on the demagnetising field [4,5]. The outer N leads are purposely extended (for simplicity here they are simply extended along the *z*-axis, but more complicated contact geometries are possible) to allow the spin accumulation to decay to zero – it is important to include in the model enough of the contacting electrical leads since only then can the boundary condition $(\nabla \mathbf{S}).\mathbf{n} = 0$ be applied correctly. The resulting *x* components of the spin accumulation for the AP and P states are shown in Figure S1b. The same material parameters for the N and F layers used in the main text are applied here using the spin mixing conductance interface approach (but with $\theta_{SHA} = 0$); additionally $\beta_D = 0.9$ [6]. The results are shown in Figure S1, where the magnetisation of the free layer is switched from the AP to the P state using a charge current density along the **z** direction of -10^{12} A/m^2 (electrons flow from the fixed to the free layer), and back to the AP state using a charge current density of $+10^{12}$ A/m^2. For simplicity the layers are not surface-exchange coupled, but interact only through the demagnetising field and the spin torque. As expected the resistance switches from a high state (AP) to a low state (P), and back to the original state. Since the demagnetising field preferentially acts to keep the layers in the AP state, the switching process is slower from the AP to the P state than vice-versa, however once the P state is achieved the shape anisotropy of the ellipse stabilises this configuration.

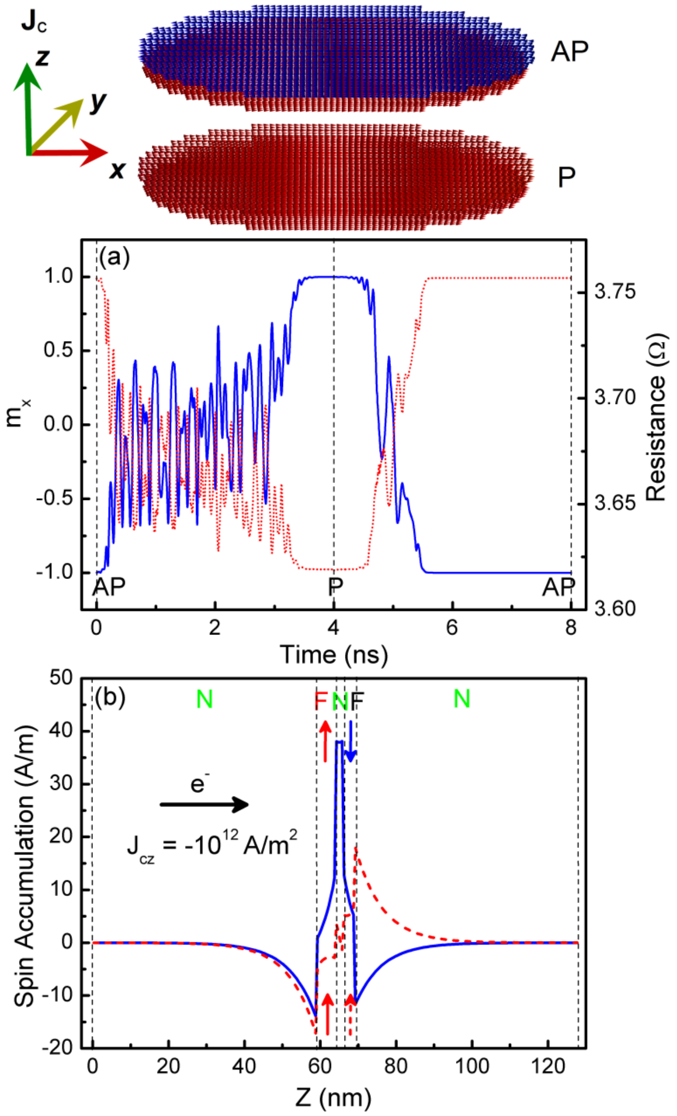

Figure S1. Current-induced magnetisation switching in a spin valve. Spin torque switching in an elliptically shaped CPP-GMR stack, showing **(a)** normalised *x* component of magnetisation (solid line) and stack resistance (dotted line) showing switching from the AP state (using $J_{cz} = -10^{12}$ A/m^2) to the P state and back (using $J_{cz} = +10^{12}$ A/m^2) to the AP state, **(b)** *x* component of spin accumulation for the AP and P configurations.

References

1. Zhang, S., Levy, P. M. & Fert, A. Mechanisms of spin-polarized current-driven magnetization switching. Phys. Rev. Lett. 88, 236601 (2002).

2. Berkov, D. V. & Miltat, J. Spin-torque driven magnetization dynamics: Micromagnetic modeling. *J. Magn. Magn. Mater.* **320**, 1238–1259 (2008).

3. Valet, T. & Fert, A. Theory of the perpendicular magnetoresistance in magnetic multilayers. *Phys. Rev. B* **48**, 7099–7113 (1993).

4. Lepadatu, S. Effective field model of roughness in magnetic nano-structures. J. Appl. Phys. 118, 243908 (2015).

5. Donahue, M. J. & McMichael, R. D. Micromagnetics on curved geometries using rectangular cells: error correction and analysis. *IEEE Trans. Mag.* **43**, 2878–2880 (2007).

6. Bass, J. & Pratt, W. P. Jr. Spin-diffusion lengths in metals and alloys, and spin-flipping at metal/metal interfaces: an experimentalist’s critical review. J. Phys. Condens. Matter 19, 183201 (2007).
